# *Sargassum* sp. extract improve hematological profile of tilapia fish ( *Oreochromis niloticus*)

**DOI:** 10.12688/f1000research.128819.2

**Published:** 2023-08-03

**Authors:** Mohamad Gazali, Irwan Effendi, Amir Husni, Nurjanah Nurjanah, Sri Wahyuni, Ronal Kurniawan

**Affiliations:** 1 Faculty of Fisheries and Marine Science, Teuku Umar University, Meulaboh, Indonesia; 2 Faculty of Fisheries and Marine, Universitas Riau, Pekanbaru, Indonesia; 3 Faculty of Agriculture, Universitas Gadjah Mada, Yogyakarta, Indonesia; 4 Faculty of Fisheries and Marine Science, Bogor Agricultural University, Bogor, Indonesia

**Keywords:** Sargassum sp. extract, hematology, A. hydrophila, fish health status

## Abstract

**Background:** Strategies to increase body resistance and prevent disease in aquaculture include using vaccines, antibiotics, and probiotics. Today, the use of antibiotics with natural ingredients is becoming a trend. One of the natural ingredients that contain high antioxidants and antibiotics is *Sargassum* sp.

**Methods:** This research was conducted from March to May 2022 at the Biotechnology Laboratory, Faculty of Fisheries and Marine, Universitas Riau, in two stages: 1) the sensitivity of extracts of *Sargassum* sp. and 2) the application of *Sargassum* sp. extract orally in tilapia ( *O. niloticus*). The parameters measured were clear zone, minimum inhibitory concentration, LD _50_ test of leaf extract of *Sargassum* sp. in tilapia ( *O. niloticus*), hemoglobin levels, hematocrit, total leukocytes, total erythrocytes, leukocyte differentiation, and survival rate. Data on hematology parameters were tabulated and analyzed using a One-Way ANOVA followed by a Student Newman Keuls (SNK) test when deemed necessary.

**Results:** The results showed that the extract of *Sargassum* sp. inhibited the growth of *Aeromonas hydrophila* bacteria with a clear zone of 6.5-15.0 mm, which is classified as resistant. At doses of 2000, 2500, and 3000 ppm, it did not cause death in fish for 96 hours (LD _50_). Hematological parameters can be a sign of the health status of fish. Tilapia given *Sargassum* sp. in different doses gave an effect between treatments, both after 30 days of rearing and post-test against *A. hydrophila* bacteria (p<0.05). The results showed that the hematology of fish fed with *Sargassum* sp. extract was in the normal or healthy range. Healthy tilapia had erythrocyte counts ranging from 1.34-2.11×10 ^6^ cells/mm ^3^, hematocrit 26.17-33.19%, hemoglobin 6.26-11.2 g/dL and total leukocytes 1.01-1.50×10 ^4^ cells/mm ^3^ and total erythrocytes 5.88-9.13×10 ^4^ cells/ mm ^3^.

**Conclusions:** A dose of 3000 ppm provided the highest health improvement against *A. hydrophila* bacterial infection.

## Introduction

Aquaculture is the answer to meeting the global nutritional needs of a growing population and ensuring food safety from aquatic sources. ^1^ Tilapia ( *Oreochromis niloticus*) is an omnivorous fish with advantages such as tolerance to different environments, high economic value, and high market demand. ^2^^,^^3^ To meet the demand, intensive aquaculture with high stocking density and artificial feed has become the standard in fish farming. ^4^ Challenges in this aquaculture include feed costs, environmental pollution, climate change, water quality, and pathogen infection, ^5^ which can reduce the productivity and profitability of aquaculture activities. ^6^^,^^7^

Motile *Aeromonas* septicaemia (MAS) caused by *Aeromonas hydrophila* is a bacterial disease that can cause mass mortality in a culture. ^7^^,^^8^ The pathogenicity of *A. hydrophila* can cause mortality in cultured fish up to 80-100% within one to two weeks. ^9^ Strategies to increase body resistance and prevent disease in cultivation including using vaccines, antibiotics, and probiotics. Antibiotic applications have been widely used to control fish diseases, including MAS. However, its use affects the environment and human health, including causing the multi-resistance of bacteria and the accumulation of these compounds in foodstuffs. ^10^^,^^11^

Understanding how specific and non-specific immune responses modulate fish health is key to increasing productivity and reducing losses in the intensive aquaculture sector. Nowadays, the use of antibiotics with natural ingredients is becoming a trend. Some immunostimulants used as additives in feed can enhance the body's defense system and thus prevent losses from disease. ^12^ In addition, enhancing the immune response with environmentally friendly materials is an effective strategy to promote sustainable cultivation.

Marine macroalgae are natural ingredients that contain high antioxidants and antibiotics. Macroalgae contain primary metabolites such as vitamins, minerals, fiber, alginate, carrageenan, and agar which are widely used as cosmetic ingredients for skin care. The brown macroalga *Sargassum* sp. has an essential function in marine ecosystems. ^13^ One of the most important functions of *Sargassum* is to provide a nursery habitat for juvenile fish and other marine animals. ^14^ The floating mats of *Sargassum* provide shelter for young fish and other animals to hide from predators, feed, and grow. ^15^ *Sargassum* sp. is a brown macroalga that grows in the mid-littoral to sublittoral zone. ^16^ *Sargassum* sp. Is mostly unexploited and has been found to contain phenols, alkaloids, triterpenoids, ^17^ saponins, and flavonoids. ^18^ According to Lee *et al.,* ^19^ the extract of *S. horneri,* an additional immunostimulant in feed, showed significant results supporting its use in white shrimp culture. In addition, the macroalga *Sargassum* sp. has been used to enhance to the immune response in several fish, including hybrid red tilapia, ^20^ tilapia, ^21^ rainbow trout, ^22^ indian major carp, ^23^ spotted scat, ^24^ and great sturgeon. ^25^ Therefore, it is necessary to research the effect of *Sargassum* sp. as a feed supplement for immune response and prevention of infection with *A.hydrophila* bacteria in tilapia ( *O. niloticus*).

## Methods

### Time and location

This research was conducted from March to May 2022 at the Biotechnology Laboratory, Faculty of Fisheries and Marine, Universitas Riau.

This research was conducted in two stages, namely 1) the sensitivity test of extracts of *Sargassum* sp. and 2) the oral administration of extracts of *Sargassum* sp. in tilapia (O *. niloticus*). The experiments were carried out within the ethical guidelines provided by the research institution and national or international regulations.

### Sensitivity test

Extraction of macroalgae *Sargassum* sp. using maceration method with ethanol solvent. A sensitivity test was performed using the Kirby Bauer disc method. To reduce the error rate, it was repeated three times. Determination of the dose used refers to the study of Ref. 26. The dose of macroalgae extract used is as follows:

Oxytetracycline antibiotics as a positive control

D1: 100% macroalgae extract equivalent to 10,000 ppm

D2: 90% macroalgae extract equivalent to 9,000 ppm

D3: 80% macroalgae extract equivalent to 8,000 ppm

D4: 70% macroalgae extract equivalent to 7,000 ppm

D5: 60% macroalgae extract equivalent to 6,000 ppm

D6: 50% macroalgae extract equivalent to 5,000 ppm

D7: 40% macroalgae extract equivalent to 4,000 ppm

D8: 30% macroalgae extract equivalent to 3,000 ppm

D9: 20% macroalgae extract equivalent to 2,000 ppm

D10: 10% macroalgae extract equivalent to 1000 ppm

The parameters measured during the study were:

1. Clear zone
2. Minimum inhibitory concentration
3. LD _50_ test of leaf extract of *Rhizophora* sp. in tilapia ( *O. niloticus*).

Observation of the inhibition zone of *Sargassum* sp. against bacteria *A. hydrophila* was conducted using the Kirby-Bauer disc method, using a 6 mm diameter blank disk. In the initial stage, a solution of *Sargassum* sp. 50 μL and oxytetracycline as a control was applied into a blank disc using a micropipette. Next, the blank disc was left for ±3 minutes to absorb the solution. It was then placed on TSA media containing *A. hydrophila* bacterial inoculant and incubated for 24 hours at 37°C. After 24 hours, the inhibition zone was observed by measuring the diameter of the clear zone formed using a calliper.

The dose used in the MIC test was based on the extract dose that produced the smallest inhibition zone to one that did not produce any. Each extract dose was added with 50 μL of bacterial suspension (bacterial density 108 CFU/mL). The solution was then homogenized and incubated for 24 hours at 37°C. The number of colonies was observed by isolating the bacteria from a solution previously incubated for 24 hours into 50 μL of TSA media and then re-incubated. After 24 hours, the bacterial colonies in the dish were counted, containing 30-300 colonies.

The LD50 toxicity test was initiated by preparing 120 tilapia fish into a container containing *Sargassum* sp. extract according to the treatment dose, referring to the MIC test. Each container contained 10 L of water, with a stocking density of 1 fish/1 L. LD50 observations was undertaken for 24-96 hours by observing the behaviour, clinical symptoms, and fish mortality reaching 50%.

### Oral administration of *Sargassum* sp. extract to tilapia ( *O. niloticus*)

Observation of the immune response was carried out using the experimental method by applying a one-factor completely randomized design (CRD) with five levels of treatment; to reduce the error rate, it was repeated three times so that 15 experimental units were needed. The treatment doses in this study were selected according to the preliminary test as follows:

NC: Negative control (feeding without *Sargassum* sp. extract and without being infected with *A. hydrophila* bacteria)

PC: Positive control (feeding without *Sargassum* sp. extract and infected with *A. hydrophila* bacteria)

F1: Feed containing *sargassum* sp. extract at a dose of 2.0 g/kg feed

F2: Feed containing *sargassum* sp. extract at a dose of 2.5 g/kg feed

F3: Feed containing *sargassum* sp. extract at a dose of 3.0 g/kg feed

### Measured parameters

The parameters measured were hemoglobin levels, hematocrit, total leukocytes, total erythrocytes, leukocyte differentiation, and survival rate.

This study used 300 fingerlings of tilapia, around 5.20±0.03 g BW. The fish specimens were selected based on their performance, indicated by active swimming, no wounds, or external parasites. Before the treatment, the fish was adapted for a week. The experiments were carried out within the ethical guidelines provided by the research institution and national or international regulations.

Macroalga *Sargassum* sp. has very potential to be used in this study because all macroalga species *Sargassum* sp. It can be used for leaves, stems, and roots/rhizoids. The macroalga were dried and crushed using a blender. Once it was smooth, it was extracted, and the products were mixed into commercial feed with the dose according to the treatment. Next, it was printed and dried before a proximate test was carried out to examine the nutritional content of the pellets.

The fish were reared in a 60 × 30 × 30 cm aquarium filled with 60 L fresh water and the fish density was 1 fish/3 L water. The fish was reared for 30 days and fed three times/day (08.00 AM, 01.00, and 06.00 PM). The total feed provided was 5% of body weight per day.

The *A. hydrophila* strain (ATCC 35654) used in this study was obtained from the Fish Quarantine in Pekanbaru, Riau, Indonesia. Fish were infected with *A. hydrophila* (0.1 mL 10 ^8^ of *A. hydrophila* culture). Negative control (Nc) were fish without any treatment, while positive control (Pc) were fish infected with *A. hydrophila* and not given *Sargassum* extract. A total of 15 fish from each treatment were studied. Blood sampling was conducted three times: at the beginning, middle, and end of the study period, with three fish from each aquarium. They were anesthetized using clove oil (5 drops/L). While they were inactive and unresponsive to touch, blood was then drawn from the tail vein by inserting a 10% moistened EDTA (Merck) syringe. Blood samples were stored in moistened EDTA vials in cold boxes filled with crushed ice. Total erythrocytes and leukocytes were counted using a Neubauer hemocytometer and then counted ^27^ and analyzed. ^28^ Hematocrit and leukocrit levels were determined using heparin capillary micro-hematocrit and centrifuged at 12,000 rpm for three minutes. To calculate the hematocrit or leukocrit level, the length of the column of packed red cells or packed white cells was measured and divided by the length of the entire column of blood (cells and plasma) and multiplied the number by 100%. Hemoglobin levels were measured using Sahli’s method. ^29^

### Statistical analyses

Data on hematology parameters (the total erythrocytes, hematocrit level, hemoglobin, total leucocytes, leucocyte differentiation, Phagocytic Index, and survival) were tabulated and analyzed using SPSS 26. Data were analyzed using a one-way ANOVA followed by a Student Newman Keuls (SNK) test if necessary.

### Ethical statement

All animals were treated according to animal welfare guidelines that have been established and approved by the Dean of the Faculty of Fisheries and Marine Science, Teuku Umar University, Prof. M. Ali Sarong; Prof. Sarong serves as the ethical committee who approved the use of vertebrae animal in these experiments with approval number: 0674/UN59.3/TU.00.01/2022.

## Results

### *A. hydrophila* bacteria sensitivity to *Sargassum* extract

The results of the sensitivity test showed that the use of Oxytetracycline and extracts of *Sargassum* sp. with doses of 100%, 90%, 80%, 70%, 60%, 50%, 40%, 30%, 20%, and 10% resulted in different inhibitory zones against the growth of *A. hydrophila* bacteria. Doses of 30-100% can inhibit the growth of *A. hydrophila* bacteria with an inhibition zone ranging from 6.5-15.0 mm, while doses of 10-20 % are no longer formed inhibition zones. More details can be seen in Table 1.

**Table 1.** Observation of the inhibition zone of macroalga extracts against *Aeromonas hydrophila* bacteria.

Macroalga extract dosage (%)	Inhibition zone (mm)
	*Sargassum* sp.
Oxytetracycline	27.3
100	15.0
90	11.5
80	10.0
70	9.7
60	9.0
50	8.3
40	7.5
30	6.5
20	0
10	0

The minimum inhibitory concentration (MIC) test was carried out based on the results of the sensitivity test of *Sargassum* sp. with a dose that produces a minimum zone of inhibition, which was then diluted to obtain 30%, 25%, and 20% doses. The MIC test aimed to determine the minimum concentration to inhibit bacterial growth. The results showed that a dose of 20-30% produced an average number of colonies capable of inhibiting the growth of *A. hydrophila* bacteria ranging from 155.33-215×10 ^8^ CFU/mL. A dose of 20% was the minimum dose to inhibit the growth of *A. hydrophila* bacteria. On the other hand, in the control treatment (0%), the number of growing bacterial colonies was uncountable. The results of the MIC test can be seen in Table 2.

**Table 2.** The number of *A. hydrophila* colonies bacteria after being given *Sargassum* sp.

Extract dosage (%)	Repetition			The average number of bacterial colonies (CFU/mL)
	1	2	3	
0	∞	∞	∞	∞
20	218	213	125	215.33×10 ^8^
25	174	177	177	179.33×10 ^8^
30	153	155	158	155.33×10 ^8^

Remarks. ∞: infinity.

**Figure 1.**
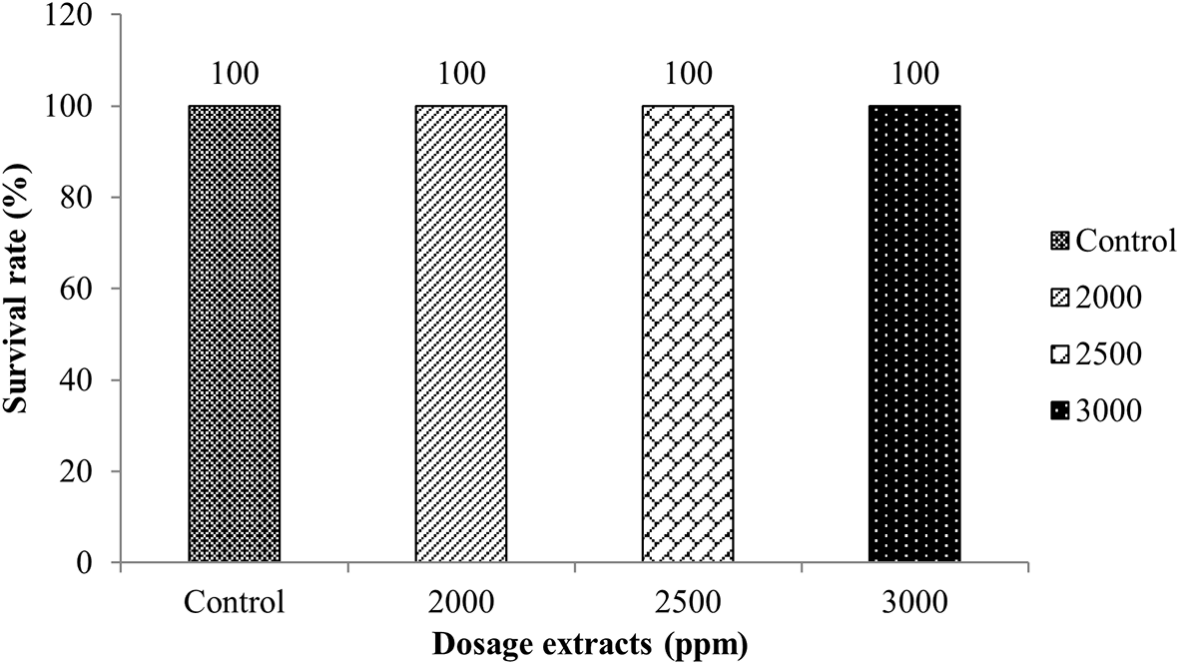
Calculation results of LD _50_ determination for 96 hours.

The LD _50_ toxicity test of macroalga extract was carried out to obtain a dose of extract that caused 50% death for 96 hours in 10 tilapia tested per aquarium. The doses used were based on the MIC test results obtained, namely 20% (2000 ppm), 25% (2500 ppm), and 30% (3000 ppm) and control. The results showed that tilapia immersion with *Sargassum* sp. did not die after 96 hours of experience, indicating that the *Sargassum* sp. was not toxic to fish. The results of the toxicity test can be seen in Figure 1.

### The effect of adding *Sargassum* sp. extract on tilapia erythrogam

Measurements of erythrocyte cells of tilapia fed a diet containing extracts of *Sargassum* sp. with different doses can be seen in Table 3. The administration of *Sargassum* sp. in the feed with different doses caused an increase in the immune response, which was shown by the increase in total erythrocytes ranging from 1.57-1.93×10 ^6^ cells/mm ^3^, when compared to fish given the feed without the addition of *Sargassum* sp. Which ranged from 1.40-1.67×10 ^6^ cells/mm ^3^ after 30 days of rearing and post-challenge test with *A. hydrophila* bacteria (p<0.05). Meanwhile, tilapia fed without *Sargassum* extract could not be examined for erythrocyte cell numbers due to mortality reaching 100%.

**Table 3.** Erythogram of tilapia given feed containing extract of *Sargassum* sp.

Observation time	Parameter	Treatment				
		NC	PC	F1	F2	F3
30 days of rearing	RBC (×10 ^6^ cell/mm ^3^)	1.40±0.04 ^a^	1.41±0.04 ^a^	1.57±0.04 ^b^	1,65±0.03 ^c^	1.76±0.03 ^d^
	Hemoglobin (g/dL)	7.20±0.20 ^a^	7.27±0.12 ^a^	7.67±0.12 ^b^	7.73±0.12 ^b^	7.93±0.12 ^b^
	Hematocrit (%)	28.33±0.58 ^a^	28.67±0.58 ^a^	29.67±0.58 ^a^	31.00±1.00 ^b^	31.67±0.58 ^b^
14 days post-challenge test	RBC (×10 ^6^ cell/mm ^3^)	1.67±0.02 ^b^	0.00±0.00 ^a^	1.70±0.02 ^c^	1.83±0.02 ^d^	1.93±0.02 ^e^
	Hemoglobin (g/dL)	7.47±0.12 ^b^	0.00±0.00 ^a^	8.00±0.20 ^c^	8,27±0.12 ^d^	8.73±0.12 ^e^
	Hematocrit (%)	29.67±0.58 ^b^	0.00±0.00 ^a^	31.00±1.00 ^c^	31.67±0.58 ^c^	31.67±0.58 ^c^

Remarks. RBC: Red blood cellconcentration; F1: Extract dose 2000 ppm; F2: 2500 ppm, F3: 3000 ppm.

Superscript letters on the same line show significantly different results between treatments (p<0.05).

The increase in total erythrocytes was in line with the increase in hemoglobin and hematocrit levels in tilapia fed with *Sargassum* sp. extract after 30 days of rearing and post-test against *A. hydrophila* bacteria (p<0.05).

### The effect of adding *Sargassum* sp. extract on tilapia leukogram

Measurement of leukocyte cells of tilapia fed a diet containing extracts of *Sargassum* sp. with different doses can be seen in Table 4. Feed containing extracts of *Sargassum* sp. increased the immune response of tilapia, indicated by an increase in total leukocytes ranging from 1.91-1.99×10 ^4^ cells/mm ^3^ when compared to the control treatment ranging from 1.88-1.89×10 ^4^ cells/mm ^3^ after 30 days of rearing (p<0.05). This range remained in normal conditions. In addition, it also affected the concentration of lymphocytes ranging from 76.67-79.33% (p<0.05). The concentration of monocytes, neutrophils, and platelets was not significantly different between treatments (p>0.05).

**Table 4.** Leukogram of tilapia after feed containing extracts of *Sargassum* sp.

Observation Time	Parameter	treatment				
		NC	PC	F1	F2	F3
30 days of rearing	WBC (×10 ^4^ cell/mm ^3^)	1.89±0.01 ^ab^	1.88±0.01 ^a^	1.91±0.01 ^b^	1.94±0.02 ^c^	1.99±0.02 ^d^
	Lymphocytes (%)	75.33±0.58 ^a^	75.67±0.58 ^ab^	76.67±0.58 ^bc^	77.67±0.58 ^c^	79.33±0.58 ^d^
	Monocytes (%)	8.67±0.58	9.00±1.00	8.33±0.58	8.00±1.00	7.67±0.58
	Neutrophil (%)	8.33±0.58	8.00±1.00	7.67±0.58	7.00±1.00	6.33±0.58
	platelets (%)	7.67±0.58	7.33±0.58	7.33±0.58	7.33±0.58	6.67±0.58
14 days post-challenge test	WBC (×10 ^4^ cell/mm ^3^)	1.92±0.01 ^b^	0.00±0.00 ^a^	2.01±0.01 ^c^	2.04±0.01 ^d^	2.10±0.02 ^e^
	Lymphocytes (%)	76.67±0.58 ^b^	0.00±0.00 ^a^	79.00±1.00 ^c^	81.33±0.58 ^d^	83.33±0.58 ^e^
	Monocyte (%)	8.00±1.00 ^c^	0.00±0.00 ^a^	7.67±0.58 ^c^	6.67±0.58 ^bc^	6.00±1.00 ^b^
	Neutrophil (%)	7.67±1.15 ^c^	0.00±0.00 ^a^	7.33±0.58 ^c^	5.67±0.58 ^b^	5.33±0.58 ^b^
	platelets (%)	7.67±0.58 ^c^	0.00±0.00 ^a^	6.00±1.00 ^b^	6.33±0.58 ^b^	5.33±0.58 ^b^

Remarks. WBC: White blood cell concentration; F1: extract dose 2000 ppm; F2: 2500 ppm, F3: 3000 ppm.

Superscript letters on the same line show significantly different results between treatments (p<0.05).

After the challenge test, the total leukocytes of fish increased between 2.01-2.10×10 ^4^ cells/mm ^3^ (p<0.05), this range was in normal conditions. The leukogram of PC could not be observed because the mortality reached 100%. Feed with extracts of *Sargassum* sp. at different doses affected the concentration of lymphocytes, monocytes, neutrophils, and platelets of tilapia (p<0.05).

### Survival of tilapia ( *O. niloticus*)

**Figure 2.**
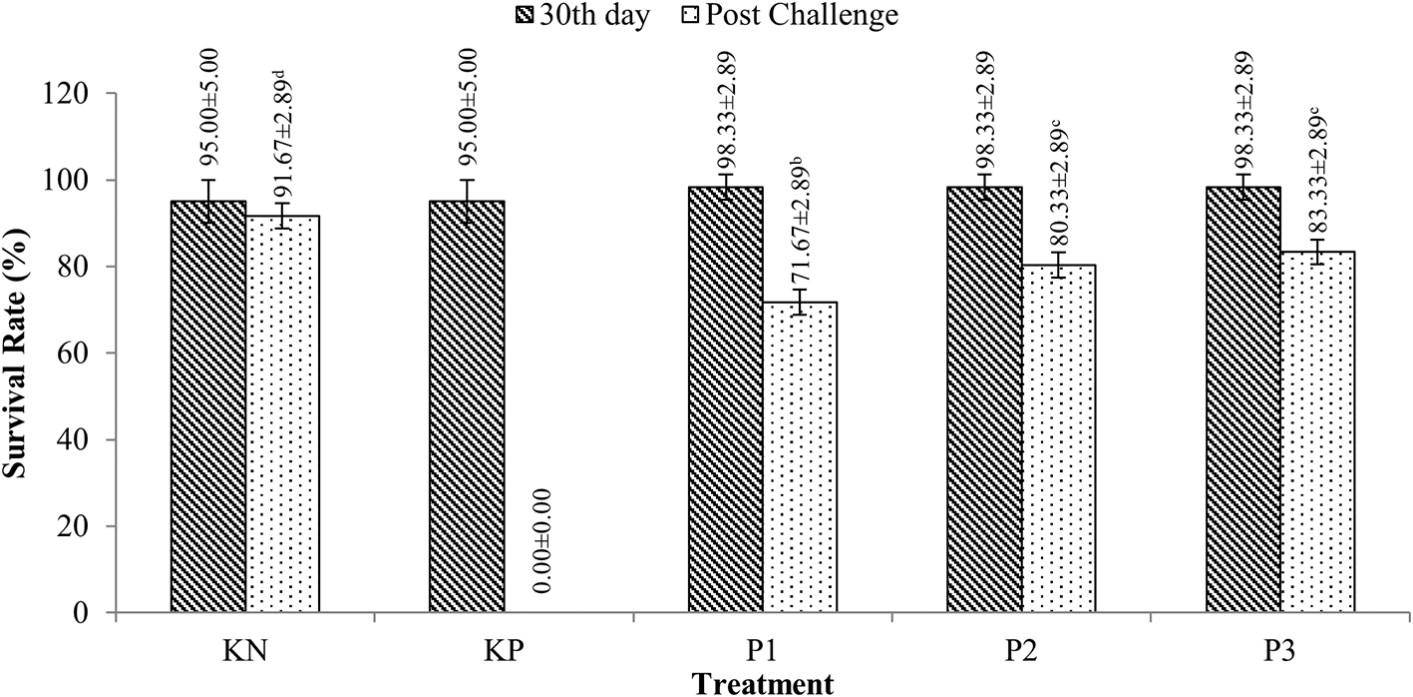
Tilapia survival.

Tilapia survivors were fed with extracts containing *Sargassum* sp. with doses ranging from 95-98.33% for 30 days of rearing (p>0.05). After the challenge test with *A. hydrophila* bacteria, tilapia survival ranged from 71.67-83.33%, while the KP (control feed and challenged *A. hydrophila*) experienced 100% mortality. More details are presented in Figure 2.

## Discussion

*Sargassum* sp extract at a dose of 100% - 30% inhibited the growth of *A. hydrophila* bacteria. This can be seen from the average inhibition zone formed for each dose. Wayne ^30^ categorizes the resulting inhibition zones into susceptible, intermediate, and resistant criteria. The CLSI criteria define the sensitivity as susceptible if the diameter of the inhibition zone formed is 21 mm, intermediate if the diameter of the inhibition zone is between 16-20 mm, and resistant if the diameter of the inhibition zone is 15 mm. This study obtained an inhibitory zone of 6.5–15 mm against *A. hydrophila* bacteria, so the overall treatment group corresponded with the resistant criteria for *Sargassum* sp. against *A. hydrophila* bacteria.

Calculation of the growth of the number of bacterial colonies of *A. hydrophila* given a solution of the extract of *Sargassum* sp. with doses of 20%, 25%, and 30% resulted in the average number of colonies capable of inhibiting the growth of *A.hydrophila* bacteria ranging from 155.33×10 ^8^ CFU/mL – 215.33×10 ^8^ CFU/mL. At a dose of 20%, the average number of bacterial colonies was 215.33×10 ^8^ CFU/mL. It could be said that the minimum dose inhibited the growth of *A.hydrophilla* bacteria. The best bacterial colony growth in inhibiting bacterial growth is 30-300 colonies. ^31^

The survival rate of tilapia against *Sargassum* sp. showed that 2000, 2500, and 3000 ppm doses did not cause fish death. This indicates that *Sargassum* sp. extract is not a toxic substance. In addition, this type of macroalga has active ingredients that can inhibit the growth of bacteria, so it is expected to be an alternative disease control in aquaculture in Indonesia.

According to Tavares-Dias *et al.* and Fagbenro *et al.,* ^32^^,^^33^ stress and nutritional imbalance can trigger changes in tilapia blood parameters. Hematological parameters are essential for assessing the health status of fish and evaluating fish physiology, feed impact, and other stressors. Environmental conditions, sex, age, feeding, and fish activity can affect hematological parameters. ^34^

In this study, the hematological parameters (erythrogram and leukogram) of tilapia that were given to the addition of *Sargassum* sp. with different doses showed an effect between treatments, both after 30 days of rearing and post-test against *A. hydrophila* bacteria (p<0.05). This indicates that the administration of *Sargassum* sp. can affect fish health. A dose of 3000 ppm leads to the highest health improvement against *A. hydrophila* bacterial infection, thought to be caused by the nutrients and secondary metabolites in the feed capable of meeting the needs in forming the fish's immune system.

Fish infected with the bacteria experience changes in the number of erythrocytes, hemoglobin, hematocrit, and total leukocytes. The results showed that the hematology of fish fed with *Sargassum* sp. extract was within the normal or healthy ranges. Healthy tilapia have erythrocyte counts ranging from 1.34-2.11×10 ^6^ cells/mm ^3^, hematocrit 26.17-33.19%, ^35^ hemoglobin 6.26-11.2 g/dL, ^36^ and total leukocytes 1.01-1.50×10 ^4^ cells/mm ^3^, ^37^ 5.88-9.13×10 ^4^ cells/mm ^3^. ^38^ Increased hemoglobin levels are associated with increased oxygen transport capacity and the body's defense mechanism against stress.

The secondary metabolite contents of *Sargassum* include vitamin C, fucoidan, and flavonoids. Vitamin C in *Sargassum* sp. can increase immunity and function and act as a fish immune system booster. ^39^ In addition, fucoidan can stimulate the immune response by producing fish immune cells. However, given its ability to activate immune cells and promote cytokine production, fucoidan may have potential as an immune-boosting supplement. ^40^ Flavonoids function as immunomodulators or substances that can affect the quality and intensity of the immune response. Flavonoids can stimulate the immune system by sending intracellular signals to cell receptors, making cell performance more active. The action of active ingredients, especially flavonoids, in stimulating the immune system is to accelerate the activation of leukocytes and macrophages so that the phagocytosis process against foreign bodies can be carried out quickly. ^41^^,^^42^

Vitamin C can increase the body's resistance to pathogens; its role in protein synthesis is necessary for immune responses and collagen biosynthesis to accelerate the wound healing process. Leukocytes, in addition to the thymus gland, spleen, and immune cells, store large concentrations of vitamin C. In stressed fish, the number of lymphocyte cells in the blood and lymphoid organs (bone marrow, lymph glands, and spleen) decreases. ^43^ Hazzuli *et al.* ^44^ stated that fish would respond to vitamin C by increasing the activity and reactivity of cellular and humoral defense cells. Besides, it can increase fish's phagocytic activity (non-specific immune response).

Macroalgae are a valuable source of secondary metabolites, which can increase immune responses and are used as immunostimulants in aquaculture fish feed. ^45^ *Sargassum* sp. extract application on immune responses has been carried out in several types of aquatic biota such as tiger prawns, ^46^ mullet, ^47^ Asian sea bass, ^48^ and rainbow trout. ^49^^,^^50^ Generally, it has been shown that *Sargassum* extract can be used as an immunostimulant.

## Conclusions

The results of the present research showed that the extract of *Sargassum* sp. was able to inhibit the growth of *A. hydrophila* bacteria with an inhibition zone (clear zone) of 6.5-15.0 mm, which is classified as resistant; at doses of 2000, 2500, and 3000 ppm it did not cause death in fish for 96 hours (LD _50_). Hematological parameters can be a sign of the health status of fish. Tilapia given *Sargassum* sp. extract at different doses showed an effect between treatments after 30 days of rearing and post-test against *A. hydrophila* bacteria (p<0.05). The results showed that the hematology of fish fed with *Sargassum* sp. was within the normal or healthy range. Healthy tilapia had erythrocyte counts ranging from 1.34-2.11×10 ^6^ cells/mm ^3^, hematocrit 26.17-33.19%, hemoglobin 6.26-11.2 g/dL, total leukocytes 1.01-1.50×10 ^4^ cells/mm ^3^ and total erythrocytes 5.88-9.13×10 ^4^ cells/mm ^3^. A dose of 3000 ppm led to the highest health improvement against *A. hydrophila* bacterial infection.

## Author roles

**Gazali M**: Conceptualization, Data Curation, Formal Analysis, Methodology, Writing – Original Draft Preparation; **Effendi I**: Data Curation, Formal Analysis, Supervision, Methodology, Writing – Original Draft Preparation; **Husni A:** Data curation, Validation, Writing-Review & Editing; **Nurjanah N:** Data Curation, Validation, Writing-Review & Editing; **Wahyuni S:** Conceptualization, Investigation, Methodology, Writing – Original Draft Preparation; **Kurniawan R:** Conceptualization, Investigation, Methodology, Software, Writing – Original Draft Preparation

## Data Availability

Zenodo: Sargassum sp. extract improve Hematological profile of Tilapia fish (Oreochromis niloticus), https://doi.org/10.5281/zenodo.7595715. ^51^
- Erytrogram, leucogram, survival.xlsx -
Figure 1. docx -
Table 1. docx - TABLE CLEAR ZONE, COUNT BACTERIA, LD50.xlsx Data are available under the terms of the Creative Commons Attribution 4.0 International license (CC-BY 4.0).
