# Supplementary material for: Sargassum sp. extract improve hematological profile of tilapia fish ( Oreochromis niloticus)
Source: F1000Res. 2024 Apr 22;12:293. Originally published 2023 Mar 16. [Version 4] doi: 10.12688/f1000research.128819.4 (PMC11137480; doi:10.12688/f1000research.128819.4)
Supplement: Peer review history

# Reviewer report: Reviewer response for version 4

**DOI:** 10.5256/f1000research.164705.r268431

**Published:** 2024-05-29

**Authors:** Malgorzata Witeska

**Affiliations:** 1 Department of Ichthyology and Biotechnology in Aquaculture, Institute of Animal Science, Warsaw University of Life Sciences, Warsaw, Poland

The Authors revised the manuscript according to the reviewer's comments.

Is the work clearly and accurately presented and does it cite the current literature?

Partly

If applicable, is the statistical analysis and its interpretation appropriate?

Partly

Are all the source data underlying the results available to ensure full reproducibility?

No

Is the study design appropriate and is the work technically sound?

Partly

Are the conclusions drawn adequately supported by the results?

Partly

Are sufficient details of methods and analysis provided to allow replication by others?

Partly

Reviewer Expertise:

Fish biology and hematology, toxicolofy and physiology

I confirm that I have read this submission and believe that I have an appropriate level of expertise to confirm that it is of an acceptable scientific standard.

# Reviewer report: Reviewer response for version 3

**DOI:** 10.5256/f1000research.161567.r240879

**Published:** 2024-03-28

**Authors:** Malgorzata Witeska

**Affiliations:** 1 Department of Ichthyology and Biotechnology in Aquaculture, Institute of Animal Science, Warsaw University of Life Sciences, Warsaw, Poland

The manuscript still requires a careful language revision.

Is the work clearly and accurately presented and does it cite the current literature?

Partly

If applicable, is the statistical analysis and its interpretation appropriate?

Partly

Are all the source data underlying the results available to ensure full reproducibility?

No

Is the study design appropriate and is the work technically sound?

Partly

Are the conclusions drawn adequately supported by the results?

Partly

Are sufficient details of methods and analysis provided to allow replication by others?

Partly

Reviewer Expertise:

Fish biology and hematology, toxicolofy and physiology

I confirm that I have read this submission and believe that I have an appropriate level of expertise to confirm that it is of an acceptable scientific standard, however I have significant reservations, as outlined above.

# Reviewer report: Reviewer response for version 2

**DOI:** 10.5256/f1000research.152629.r237564

**Published:** 2024-01-17

**Authors:** Malgorzata Witeska

**Affiliations:** 1 Department of Ichthyology and Biotechnology in Aquaculture, Institute of Animal Science, Warsaw University of Life Sciences, Warsaw, Poland

The Authors performed a study of the antibiotic properties of *Sargassum* *algae* extract against *Aeromonas hydrophila* and toxicity of this alga as feed supplement to Nile tilapia. The results showed that concentrated extract of *Sargassum* had an antibiotic effect (however, weaker that oxytetracycline), addition of *Sargassum* as a feed supplement produced no toxic effect, and considerably increased (in a concentration-related way) fish survival after *Aeromonas hydrophila* challenge. This make Sargassum a potentially valuable antibacterial alternative or additive. However, I have some comments the intention of which is improving the quality of the work:

 1. Explain, please, convincingly why no negative control (with 0% extract and only ethanol at 100% level) was not included in the MIC evaluation? This makes the results incomplete - we do not know what was the contribution of ethanol in bacterial growth inhibition.

 2. Explain, please, how water quality was measured and maintained during the experiment? At 5% feeding rate and high stocking density water quality is supposed to deteriorate fast due to accumulation of uneaten food and feces.

 3. In the paragraph Statistical analyses, "leukocyte differentiation" (which probably means: leukocyte differential count - the results of which are shown in Table 4) and Phagocytic Index (the results of which are not shown) are mentioned. No methods, however, of measurement of these parameters are given. Please, add.

 4. Were dte data tested for ANOVA preconditions: normality of distribution and homogeneity of variance before the use of ANOVA?

 5. Tables 3 and 4: titles should contain "Red blood parameters" and "White blood parameters" instead of "erythrogram" and "leukogram", and the 0 values for the parameters should be removed from the PC post-challenge since the values of the parameters were not "0" but no values were obtained due to the death of all fish.

 6. The manuscript requires a careful language revision.

Is the work clearly and accurately presented and does it cite the current literature?

Partly

If applicable, is the statistical analysis and its interpretation appropriate?

Partly

Are all the source data underlying the results available to ensure full reproducibility?

No

Is the study design appropriate and is the work technically sound?

Partly

Are the conclusions drawn adequately supported by the results?

Partly

Are sufficient details of methods and analysis provided to allow replication by others?

Partly

Reviewer Expertise:

Fish biology and hematology, toxicolofy and physiology

I confirm that I have read this submission and believe that I have an appropriate level of expertise to state that I do not consider it to be of an acceptable scientific standard, for reasons outlined above.

# Reviewer report: Reviewer response for version 2

**DOI:** 10.5256/f1000research.152629.r193687

**Published:** 2023-09-25

**Authors:** Wahidatul Husna Zuldin

**Affiliations:** 1 Borneo Marine Research Institute, Universiti Malaysia Sabah, Kota Kinabalu, Sabah, Malaysia

The revised write-up are acceptable and recommended for publication.

Is the work clearly and accurately presented and does it cite the current literature?

Yes

If applicable, is the statistical analysis and its interpretation appropriate?

Yes

Are all the source data underlying the results available to ensure full reproducibility?

No source data required

Is the study design appropriate and is the work technically sound?

Yes

Are the conclusions drawn adequately supported by the results?

Yes

Are sufficient details of methods and analysis provided to allow replication by others?

Partly

Reviewer Expertise:

Seaweed Culture, Macroalgal Extraction, Microalgae Culture, Seaweed Ecology.

I confirm that I have read this submission and believe that I have an appropriate level of expertise to confirm that it is of an acceptable scientific standard.

# Reviewer report: Reviewer response for version 1

**DOI:** 10.5256/f1000research.141444.r166807

**Published:** 2023-06-08

**Authors:** Wahidatul Husna Zuldin

**Affiliations:** 1 Borneo Marine Research Institute, Universiti Malaysia Sabah, Kota Kinabalu, Sabah, Malaysia

Abstract

 The abstract was clearly written. Easy to comprehend with sufficient information on the research background, applied methods, and data produced from the study.

Introduction

 The introduction section was nicely written. However, the author is suggested to add more on past studies related to the relationship between macroalgal extract and fish hematological profile.

Methods

 Please clarify what features of *Sargassum* sp. were used in the study. Is it include the rhizoids/roots?

 When does the author state *Sargassum *sp., and what potential species were used in the study?

 Also, further, explain the extraction method. What solvent was used for the seaweed extraction?

Results and Discussion

 The results section was clearly presented. Great work on that. The data are highly useful for the tilapia industry. The discussion section was nicely written with a recent literature review included to support the findings.

Conclusions

 The conclusions are relevant and clearly stated.

 Overall, the article is well written with minor revisions required.

Is the work clearly and accurately presented and does it cite the current literature?

Yes

If applicable, is the statistical analysis and its interpretation appropriate?

Yes

Are all the source data underlying the results available to ensure full reproducibility?

No source data required

Is the study design appropriate and is the work technically sound?

Yes

Are the conclusions drawn adequately supported by the results?

Yes

Are sufficient details of methods and analysis provided to allow replication by others?

Partly

Reviewer Expertise:

Seaweed Culture, Macroalgal Extraction, Microalgae Culture, Seaweed Ecology.

I confirm that I have read this submission and believe that I have an appropriate level of expertise to confirm that it is of an acceptable scientific standard, however I have significant reservations, as outlined above.
